# Supplementary material for: Cognitive Function Trajectories and Factors among Chinese Older Adults with Subjective Memory Decline: CHARLS Longitudinal Study Results (2011–2018)
Source: Int J Environ Res Public Health. 2022 Dec 13;19(24):16707. doi: 10.3390/ijerph192416707 (PMC9778675; doi:10.3390/ijerph192416707)
Supplement: Supplementary file 1 [file ijerph-19-16707-s001.zip › ijerph-2057609-supplementary.pdf]

Supplementary Materials

Table S1. Results of the unconditional Parallel Process Curvilinear Latent Growth Curve Models (Nonlinear PP-LGCM).

	Global cognition			IADL			Fit indexes	
n=1465	**Estimate**	**SE**	**t**	**Estimate**	**SE**	**t**	χ^2 (df)	**54.808(22)**
**Intercept**	**21.539*****	1.114	19.335	**5.507*****	0.033	165.305	CFI	0.989
Intercept variance	**7.502*****	0.546	13.732	**0.853*****	0.065	13.129	TLI	0.986
**Slope**	**-0.663****	0.204	-3.249	**0.053*****	0.006	8.531	RMSEA	0.032
Slope variance	0.038	0.020	1.879	**0.016*****	0.003	4.973	SRMR	0.031
**Correlation**	**0.402*****	0.071	5.680	**0.039****	0.009	4.159		
	**Orientation**			**IADL**			**Fit indexes**	
n=1465	**Estimate**	**SE**	**t**	**Estimate**	**SE**	**t**	χ^2 (df)	**64.510(22)**
**Intercept**	**5.637*****	0.291	19.368	**5.507*****	0.033	165.320	CFI	0.981
Intercept variance	**0.442*****	0.037	11.966	**0.853*****	0.065	13.139	TLI	0.975
**Slope**	**-0.117****	0.044	-2.675	**0.053*****	0.006	8.530	RMSEA	0.036
Slope variance	**0.004****	0.001	2.908	**0.016*****	0.003	5.007	SRMR	0.035
**Correlation**	-0.001	0.005	-0.221	**0.039*****	0.009	4.126		
	**Episodic memory**			**IADL**			**Fit indexes**	
n=1465	**Estimate**	**SE**	**t**	**Estimate**	**SE**	**t**	χ^2 (df)	**51.976 (22)**
**Intercept**	**9.857*****	0.687	14.342	**5.507*****	0.033	165.278	CFI	0.986
Intercept variance	**2.749*****	0.243	11.326	**0.854*****	0.065	13.149	TLI	0.982
**Slope**	**-0.276***	0.131	-2.104	**0.053*****	0.006	8.533	RMSEA	0.031
Slope variance	0.020	0.011	1.848	**0.016*****	0.003	4.932	SRMR	0.028
**Correlation**	**0.141*****	0.036	3.960	**0.039*****	0.009	4.156		
	**Calculation**			**IADL**			**Fit indexes**	

n=1465	Estimate	SE	t	Estimate	SE	t	χ² (df)	33.593 (22)
**Intercept**	**4.741*****	0.399	11.880	**5.507*****	0.033	165.281	CFI	0.993
Intercept variance	**0.818*****	0.080	10.195	**0.852*****	0.065	13.107	TLI	0.991
**Slope**	**-0.198****	0.073	-2.709	**0.053*****	0.006	8.533	RMSEA	0.019
Slope variance	0.004	0.003	1.188	**0.016*****	0.003	4.931	SRMR	0.026
**Correlation**	0.011	0.011	1.016	**0.040*****	0.010	4.183		
	**Constructability**			**IADL**			**Fit indexes**	
n=1465	**Estimate**	**SE**	**t**	**Estimate**	**SE**	**t**	χ² (df)	**57.171 (22)**
**Intercept**	**1.308*****	0.119	11.027	**5.507*****	0.033	165.306	CFI	0.978
Intercept variance	**0.040*****	0.006	7.325	**0.852*****	0.065	13.116	TLI	0.972
**Slope**	**-0.072****	0.022	-3.350	**0.053*****	0.006	8.527	RMSEA	0.033
Slope variance	<0.001	<0.001	0.226	**0.016*****	0.003	4.908	SRMR	0.028
**Correlation**	0.001	0.001	1.240	**0.040*****	0.010	4.184		

	**Global cognition**			**Depression**			**Fit indexes**	
n=1462	**Estimate**	**SE**	**t**	**Estimate**	**SE**	**t**	χ² (df)	**140.430 (22)**
**Intercept**	**17.779*****	0.262	67.736	**7.947*****	0.134	59.445	CFI	0.968
Intercept variance	**6.785*****	0.646	10.507	**17.535*****	0.986	17.787	TLI	0.959
**Slope**	**-0.130***	0.051	-2.554	**0.035***	0.017	2.038	RMSEA	0.061
Slope variance	0.016	0.034	0.465	0.050	0.028	1.814	SRMR	0.036
**Correlation**	**0.427*****	0.113	3.775	0.014	0.104	0.137		
	**Orientation**			**Depression**			**Fit indexes**	
n=1462	**Estimate**	**SE**	**t**	**Estimate**	**SE**	**t**	χ² (df)	**126.624 (22)**
**Intercept**	**4.565*****	0.066	68.879	**7.944** ***	0.134	59.440	CFI	0.964

Intercept variance	**0.427*****	0.042	10.222	**17.530*****	0.985	17.794	TLI	0.955
**Slope**	**-0.028****	0.011	-2.616	**0.035***	0.017	2.041	RMSEA	0.057
Slope variance	0.003	0.002	1.827	0.046	0.028	1.668	SRMR	0.041
**Correlation**	0.001	0.006	0.090	0.019	0.104	0.185		
	**Episodic memory**			**Depression**			**Fit indexes**	
n=1465	**Estimate**	**SE**	**t**	**Estimate**	**SE**	**t**	χ^2 (df)	**122.735 (22)**
**Intercept**	**8.392*****	0.173	48.577	**7.944** ***	0.134	59.438	CFI	0.965
Intercept variance	**2.428*****	0.289	8.386	**17.530*****	0.985	17.795	TLI	0.955
**Slope**	-0.020	0.037	-0.541	**0.035***	0.017	2.050	RMSEA	0.056
Slope variance	0.006	0.017	0.333	0.047	0.028	1.712	SRMR	0.031
**Correlation**	**0.164****	0.055	2.969	0.018	0.104	0.172		
	**Calculation**			**Depression**			**Fit indexes**	
n=1465	**Estimate**	**SE**	**t**	**Estimate**	**SE**	**t**	χ^2 (df)	**91.105 (22)**
**Intercept**	**3.917*****	0.089	43.881	**7.943*****	0.134	59.429	CFI	0.971
Intercept variance	**0.811*****	0.081	10.052	**17.521*****	0.985	17.784	TLI	0.964
**Slope**	**-0.068*****	0.015	-4.378	**0.035***	0.017	2.047	RMSEA	0.046
Slope variance	0.005	0.003	1.415	0.046	0.028	1.647	SRMR	0.031
**Correlation**	0.009	0.011	0.784	0.021	0.104	0.206		
	**Constructability**			**Depression**			**Fit indexes**	
n=1465	**Estimate**	**SE**	**t**	**Estimate**	**SE**	**t**	χ^2 (df)	**95.218 (22)**
**Intercept**	**0.914*****	0.022	40.882	**7.945*****	0.134	59.434	CFI	0.968
Intercept variance	**0.042*****	0.005	8.424	**17.520*****	0.986	17.775	TLI	0.959
**Slope**	**-0.019*****	0.004	-4.887	**0.035***	0.017	2.032	RMSEA	0.048
Slope variance	<0.001	<0.001	0.959	0.046	0.028	1.653	SRMR	0.031

**Correlation**	<0.001	0.001	0.340	0.022	0.104	0.210

* *p*<0.05, ** *p*<0.01, *** *p*<0.001. Instrumental activities of daily living (IADL).
Unconditional Parallel Process LGCM (PP-LGCM), to investigate the relationship between GC, IADL, and DP across 4 waves.

Table S2. Results of the unconditional Parallel Process linear Latent Growth Curve Models (Linear PP-LGCM).

	**Global cognition**			**IADL**			**Fit indexes**		**Global cognition on IADL** [a]		
n=1462	**Estimate**	**SE**	**t**	**Estimate**	**SE**	**t**	χ^2 (df)	**67.886(22)**		**β**	**SE**
**Intercept**	**22.451*****	1.631	13.769	**5.475*****	0.035	156.302	CFI	0.984	$I_{(IADL)} \rightarrow I_{(GC)}$	**-0.393*****	0.092
Intercept variance	**6.708*****	0.679	9.877	**0.774*****	0.078	9.930	TLI	0.980	$S_{(IADL)} \rightarrow I_{(GC)}$	0.108	0.118
**Slope**	**-1.118***	0.434	-2.573	**0.064*****	0.008	7.779	RMSEA	0.038	$I_{(IADL)} \rightarrow S_{(GC)}$	**0.510***	0.240
Slope variance	0.036	0.040	0.892	**0.022*****	0.005	4.484	SRMR	0.021	$S_{(IADL)} \rightarrow S_{(GC)}$	**-0.807****	0.270
**Correlation**	**0.552*****	0.131	4.227	**0.046****	0.015	3.031					
	**Orientation**			**IADL**			**Fit indexes**		**Orientation on IADL** [a]		
n=1462	**Estimate**	**SE**	**t**	**Estimate**	**SE**	**t**	χ^2 (df)	**74.607(22)**		**β**	**SE**
**Intercept**	**5.912*****	0.445	13.280	**5.477*****	0.035	156.319	CFI	0.976	$I_{(IADL)} \rightarrow I_{(OR)}$	**-0.413*****	0.097
Intercept variance	**0.431*****	0.049	8.815	**0.777****	0.078	9.948	TLI	0.969	$S_{(IADL)} \rightarrow I_{(OR)}$	0.167	0.123
**Slope**	**-0.194***	0.086	-2.245	**0.064*****	0.008	7.779	RMSEA	0.040	$I_{(IADL)} \rightarrow S_{(OR)}$	0.325	0.168
Slope variance	**0.006***	0.002	2.570	**0.022*****	0.005	4.553	SRMR	0.025	$S_{(IADL)} \rightarrow S_{(OR)}$	**-0.510****	0.193

**Correlation**	-0.002	0.008	-0.265	**0.045****	0.015	2.973					
	**Episodic memory**			**IADL**			**Fit indexes**		**Episodic memory on IADL** [a]		
n=1462	**Estimate**	**SE**	**t**	**Estimate**	**SE**	**t**	χ^2 (df)	**56.966(22)**		**β**	**SE**
**Intercept**	**10.108*****	0.927	10.907	**5.447*****	0.035	156.281	CFI	0.983	$I_{(IADL)} \rightarrow I_{(EM)}$	**-0.278****	0.094
Intercept variance	**2.509*****	0.304	8.239	**0.774*****	0.078	9.935	TLI	0.978	$S_{(IADL)} \rightarrow I_{(EM)}$	0.014	0.119
**Slope**	-0.463	0.244	-1.895	**0.064*****	0.008	7.780	RMSEA	0.033	$I_{(IADL)} \rightarrow S_{(EM)}$	0.336	0.221
Slope variance	0.027	0.019	1.449	**0.022*****	0.005	4.445	SRMR	0.022	$S_{(IADL)} \rightarrow S_{(EM)}$	**-0.550***	0.266
**Correlation**	**0.176****	0.060	2.929	**0.046****	0.015	3.048					
	**Calculation**			**IADL**			**Fit indexes**		**Calculation on IADL** [a]		
n=1462	**Estimate**	**SE**	**t**	**Estimate**	**SE**	**t**	χ^2 (df)	**57.265(22)**		**β**	**SE**
**Intercept**	**4.949*****	0.560	8.838	**5.447*****	0.035	156.285	CFI	0.979	$I_{(IADL)} \rightarrow I_{(CA)}$	**-0.243***	0.101
Intercept variance	**0.802*****	0.102	7.827	**0.770*****	0.078	9.869	TLI	0.974	$S_{(IADL)} \rightarrow I_{(CA)}$	0.084	0.125
**Slope**	**-0.327***	0.144	-2.266	**0.064*****	0.008	7.785	RMSEA	0.033	$I_{(IADL)} \rightarrow S_{(CA)}$	0.472	0.305
Slope variance	0.004	0.006	0.622	**0.022*****	0.005	4.441	SRMR	0.025	$S_{(IADL)} \rightarrow S_{(CA)}$	**-0.762***	0.378
**Correlation**	0.014	0.019	0.716	**0.047****	0.015	3.091					
	**Constructability**			**IADL**			**Fit indexes**		**Constructability on IADL** [a]		
n=1462	**Estimate**	**SE**	**t**	**Estimate**	**SE**	**t**	χ^2 (df)	**67.335(22)**		**β**	**SE**
**Intercept**	**1.488*****	0.208	7.161	**5.477*****	0.035	156.309	CFI	0.971	$I_{(IADL)} \rightarrow I_{(CO)}$	**-0.542****	0.148
Intercept variance	**0.035*****	0.009	4.060	**0.773*****	0.078	9.905	TLI	0.964	$S_{(IADL)} \rightarrow I_{(CO)}$	**0.366***	0.177
**Slope**	**-0.133****	0.049	-2.711	**0.064*****	0.008	7.770	RMSEA	0.038	$I_{(IADL)} \rightarrow S_{(CO)}$	0.877	0.451
Slope variance	<0.001	<0.001	-0.410	**0.105*****	0.005	4.439	SRMR	0.023	$S_{(IADL)} \rightarrow S_{(CO)}$	**-1.164***	0.539
**Correlation**	0.002	0.002	1.379	**0.046****	0.015	3.061					
	**Global cognition**			**Depression**			**Fit indexes**		**Global cognition on Depression** [a]		
n=1462	**Estimate**	**SE**	**t**	**Estimate**	**SE**	**t**	χ^2 (df)	**152.030(22)**		**β**	**SE**

**Intercept**	**18.087*****	0.551	32.817	**8.020** ***	0.142	56.670	CFI	0.965	$I_{(DP)} \rightarrow I_{(GC)}$	**-0.446*****	0.099
Intercept variance	**5.035***	1.991	2.529	**17.526*****	1.147	15.277	TLI	0.955	$S_{(DP)} \rightarrow I_{(GC)}$	0.405	0.292
**Slope**	-0.233	0.158	-1.475	0.011	0.023	0.493	RMSEA	0.064	$I_{(DP)} \rightarrow S_{(GC)}$	0.008	0.279
Slope variance	-0.073	0.176	-0.413	0.044	0.044	0.997	SRMR	0.034	$S_{(DP)} \rightarrow S_{(GC)}$	-1.319	0.698
**Correlation**	0.865	0.562	1.540	0.021	0.167	0.128					
	**Orientation**			**Depression**			**Fit indexes**		**Orientation on Depression** [a]		
n=1462	**Estimate**	**SE**	**t**	**Estimate**	**SE**	**t**	χ^2 (df)	**134.536(22)**		**β**	**SE**
**Intercept**	**4.617*****	0.144	32.113	**8.017** ***	0.142	56.655	CFI	0.962	$I_{(DE)} \rightarrow I_{(OR)}$	**-0.419***	0.208
Intercept variance	**0.377****	0.121	3.119	**17.469*****	1.145	15.252	TLI	0.951	$S_{(DE)} \rightarrow I_{(OR)}$	0.507	0.345
**Slope**	-0.043	0.033	-1.311	0.012	0.023	0.504	RMSEA	0.059	$I_{(DE)} \rightarrow S_{(OR)}$	0.147	0.379
Slope variance	0.002	0.007	0.342	0.038	0.044	0.866	SRMR	0.036	$S_{(DE)} \rightarrow S_{(OR)}$	-1.031	0.600
**Correlation**	0.009	0.027	0.344	0.037	0.167	0.224					
	**Episodic memory**			**Depression**			**Fit indexes**		**Episodic memory on Depression** [a]		
n=1462	**Estimate**	**SE**	**t**	**Estimate**	**SE**	**t**	χ^2 (df)	**126.921(22)**		**β**	**SE**
**Intercept**	**8.584*****	0.406	21.167	**8.017** ***	0.141	56.658	CFI	0.963	$I_{(DE)} \rightarrow I_{(EM)}$	**-0.430****	0.129
Intercept variance	1.744	0.954	1.828	**17.468*****	1.145	15.253	TLI	0.954	$S_{(DE)} \rightarrow I_{(EM)}$	0.444	0.367
**Slope**	-0.085	0.127	-0.673	0.012	0.023	0.501	RMSEA	0.057	$I_{(DE)} \rightarrow S_{(EM)}$	-0.001	0.354
Slope variance	-0.039	0.098	-0.394	0.038	0.044	0.852	SRMR	0.032	$S_{(DE)} \rightarrow S_{(EM)}$	-1.392	0.896
**Correlation**	0.351	0.292	1.200	0.038	0.167	0.230					
	**Calculation**			**Depression**			**Fit indexes**		**Calculation on Depression** [a]		
n=1462	**Estimate**	**SE**	**t**	**Estimate**	**SE**	**t**	χ^2 (df)	**111.428(22)**		**β**	**SE**
**Intercept**	**4.013*****	0.147	27.274	**8.016** ***	0.142	56.646	CFI	0.963	$I_{(DE)} \rightarrow I_{(CA)}$	**-0.246****	0.083
Intercept variance	**0.765*****	0.135	5.655	**17.443*****	1.145	15.228	TLI	0.953	$S_{(DE)} \rightarrow I_{(CA)}$	0.204	0.252
**Slope**	**-0.104***	0.041	-2.545	0.012	0.023	0.505	RMSEA	0.053	$I_{(DE)} \rightarrow S_{(CA)}$	0.078	0.251

Slope variance	0.003	0.010	0.254	0.036	0.044	0.812	SRMR	0.035	$S_{(DE)} \rightarrow S_{(CA)}$	-0.832	0.689
**Correlation**	0.020	0.032	0.631	0.045	0.167	0.268					
	**Constructability**			**Depression**			**Fit indexes**		**Constructability on Depression** [a]		
n=1462	**Estimate**	**SE**	**t**	**Estimate**	**SE**	**t**	χ^2 (df)	**107.236(22)**		**β**	**SE**
**Intercept**	**0.939*****	0.039	24.252	**8.018*****	0.142	56.654	CFI	0.963	$I_{(DE)} \rightarrow I_{(CO)}$	**-0.387*****	0.090
Intercept variance	**0.038*****	0.009	4.065	**17.471*****	1.147	15.229	TLI	0.953	$S_{(DE)} \rightarrow I_{(CO)}$	0.253	0.273
**Slope**	**-0.026****	0.008	-3.267	0.011	0.023	0.493	RMSEA	0.051	$I_{(DE)} \rightarrow S_{(CO)}$	0.135	0.203
Slope variance	<0.001	<0.001	0.619	0.039	0.044	0.882	SRMR	0.032	$S_{(DE)} \rightarrow S_{(CO)}$	-0.616	0.588
**Correlation**	0.001	0.002	0.413	0.037	0.168	0.220					

[a] Standardized score. Bold: statistically significant (* p<0.05, ** p<0.01, *** p<0.001).
I: Intercept; S: Slop; GC: Global Cognition; IADL: Instrumental Activities of Daily Living; DP: Depression; OR: Orientation; EM: Episodic Memory; CA: Calculation; CO: Constructability.
Unconditional Parallel Process LGCM (PP-LGCM), to investigate the relationship between global cognition, IADL, and depression across 4 waves

Table S3. Summary table of findings in relation to the trajectory hypotheses.

	Initial Level		Change Rate	
	**Protective factors**	**Risk factors**	**Protective factors**	**Risk factors**
**Global cognition**	High level of education	Advanced age	High level of education	Advanced age
	Married with a spouse	Lower IADL ability	High IADL ability	Lower IADL ability
		Depression		Rapid decrease of IADL ability
				Rapid increase of Depression levels
**Orientation**	High level of education	Depression		Rapid decrease of IADL ability
		Lower IADL ability		Rapid increase of Depression levels
		Depression		
**Episodic memory**	High level of education	Advanced age	High level of education	Rapid decrease of IADL ability
	Female	Lower IADL ability		Rapid increase of Depression levels
	Married with a spouse	Depression		
	Cardio and cerebral diseases			
**Calculation**	High level of education	Lower IADL ability		Rapid decrease of IADL ability
	Male	Depression		
**Constructability**	High level of education	Lower IADL ability		Rapid decrease of IADL ability
	Longer night sleeping time	Depression		

Table S4. Characteristics of the attrition and the analytic sample on the baseline

**Variables**	**Attrition (N=4906)**	**Analytic sample (N=1465)**	**P Value**
**Age**, Mean ± SD	69.44 ± 7.47	65.46 ± 4.56	< 0.001
**Gender**			< 0.001
Male, N (%)	2237 (45.64%)	884 (60.34%)	
Female, N (%)	2664 (54.36%)	581 (39.66%)	
**Education**			< 0.001
Illiterate, N (%)	2304 (47.08%)	165 (11.26%)	
Primary school or below, N (%)	1957 (39.99%)	863 (58.91%)	
Middle school, N (%)	409 (8.36%)	289 (19.73%)	
High school or above, N (%)	224 (4.58%)	148 (10.10%)	
**Smoking**			< 0.001
Yes, N (%)	1972 (40.34%)	706 (48.19%)	
No, N (%)	2916 (59.66%)	759 (51.81%)	
**Drinking**			< 0.001
≥1 time/month, N (%)	419 (10.85%)	432 (29.49%)	
Drink but <1 time /month, N (%)	378 (9.79%)	121 (8.26%)	
None, N (%)	3066 (79.37%)	912 (62.25%)	
**Marital status**			< 0.001
Married with spouse present	3445 (70.23%)	1243 (84.85%)	
Without spouse present	1460 (29.77%)	222 (15.15%)	
**Number of chronic diseases**			0.243
Without chronic diseases	1155 (23.59%)	348 (23.75%)	
1-3 chronic diseases	3163 (64.60%)	921 (62.87%)	
4-6 chronic diseases	549 (11.21%)	182 (12.42%)	
≥ 7 chronic diseases	29 (0.59%)	14 (0.96%)	
**Night sleeping time (hours),** Mean ± SD	6.04 ± 2.14	6.17 ± 1.79	0.036
**Napping time (minutes),** Mean ± SD	33.96 ± 44.44	36.25 ± 43.17	0.086
**Cognitive functions**			
Global cognition, Mean ± SD	10.25 ± 5.60	15.59 ± 4.22	< 0.001
Orientation, Mean ± SD	3.31 ± 1.39	4.09 ± 1.09	< 0.001
Episodic memory, Mean ± SD	5.66 ± 3.13	7.24 ± 2.95	< 0.001
Calculation, Mean ± SD	2.57 ± 1.89	3.50 ± 1.74	< 0.001
Constructability, Mean ± SD	0.42 ± 0.49	0.76 ± 0.43	< 0.001
**IADL**, Mean ± SD	7.07 ± 3.78	5.55 ± 1.51	< 0.001
**Depression**, Mean ± SD	10.16 ± 6.59	8.52 ± 6.13	< 0.001

Table S5. Results of the univariate and conditional linear Latent Growth Curve Models among participants without SMD.

Univariate LGCM

	**Mean**		**Variance**			**Fit indexes**					
n=374	**Intercept**	**Slope**	**Intercept**	**Slope**	**Correlation**	χ^2	**df**	**CFI**	**TLI**	**RMSEA**	**SRMR**
**Global cognition**	**17.710*****	**-0.286*****	**7.141*****	0.100	0.361	10.053	5	0.988	0.985	0.052	0.058
**Orientation**	**4.530*****	**-0.050*****	**0.256*****	**-0.005***	**0.027****	**17.981****	5	0.958	0.949	0.083	0.089
**Episodic memory**	**8.498*****	**-0.124*****	**3.894*****	**0.097****	-0.004	**18.508****	5	0.953	0.943	0.085	0.038
**Calculation**	**3.813*****	**-0.077*****	0.276	-0.001	0.062	**11.828***	5	0.932	0.918	0.060	0.036
**Constructability**	**0.883*****	**-0.033*****	0.006	<0.001	0.003	8.549	5	0.923	0.908	0.044	0.045

B. Conditional LGCM

	**Global cognition** [a]		**Orientation** [a]		**Episodic memory** [a]		**Calculation** [a]		**Constructability** [a]	
N = 326	**β**	**SE**	**β**	**SE**	**β**	**SE**	**β**	**SE**	**β**	**SE**
**Intercept**	**6.731*****	0.645	**8.031*****	0.996	**4.315*****	0.476	**7.309****	2.522	**7.653***	3.288
Intercept variance	**0.593*****	0.085	**0.460*****	0.116	**0.679*****	0.083	0.448	0.355	-0.499	-
**Slope**	**-1.491****	0.511	**-**	-	**-0.956***	0.433	-2.198	2.164	-10.964	347.691
Slope variance	**0.568***	0.224	**-**	-	**0.630****	0.194	**-0.343**	-	-14.570	-
**Correlation**	0.163	0.388	**-**	-	-0.080	0.275	**-**	-	**-**	-
**Intercept on baseline**										
Age	**-0.157***	0.071	-0.109	0.068	-0.127	0.075	-0.146	0.141	-0.063	0.143
Gender	0.069	0.089	-0.099	0.085	0.181	0.094	-0.182	0.176	-0.051	0.178
≤ Primary school	**-0.183***	0.072	0.037	0.067	**-0.298*****	0.075	0.108	0.138	0.226	0.164
Middle school	**0.341*****	0.070	**0.166***	0.070	**0.249****	0.074	**0.443***	0.181	**0.683***	0.293
≥ High school	**0.355*****	0.075	**0.300*****	0.075	**0.321*****	0.079	0.131	0.150	**0.679***	0.294
Smoking status	-0.103	0.083	**-0.253****	0.081	-0.014	0.087	-0.153	0.164	-0.081	0.167
Drinking status	0.013	0.077	0.005	0.073	0.014	0.080	-0.005	0.146	-0.037	0.153
Marriage	-0.035	0.143	0.067	0.151	-0.041	0.151	-0.081	0.302	-0.002	0.347
Night sleeping time	0.076	0.115	-0.065	0.123	0.132	0.120	-0.041	0.240	-0.116	0.280

Napping time	0.073	0.106	0.090	0.114	0.038	0.112	0.075	0.220	0.172	0.262
CCVD	0.009	0.168	**0.495****	0.148	-0.034	0.177	-0.334	0.329	0.176	0.406
IADL	**-0.240***	0.118	**-0.299***	0.127	-0.178	0.127	-0.182	0.254	0.166	0.300
Depression	-0.068	0.108	-0.033	0.117	-0.030	0.115	-0.147	0.225	-0.275	0.271
**Slope on baseline**										
Age	-0.137	0.111	-	-	-0.211	0.113	0.009	0.210	-0.953	30.194
Gender	0.106	0.134	-	-	0.002	0.133	0.381	0.437	0.155	5.161
≤ Primary school	0.108	0.106	-	-	0.209	0.110	-0.287	0.331	-0.624	66.563
Middle school	0.092	0.107	-	-	0.113	0.107	-0.103	0.227	-2.900	91.804
≥ High school	**0.300***	0.122	-	-	0.184	0.113	0.569	0.559	-0.041	1.908
Smoking status	0.108	0.106	-	-	-0.186	0.126	0.327	0.385	2.102	66.563
Drinking status	-0.064	0.113	-	-	0.002	0.113	-0.219	0.296	-0.116	3.886
Marriage	0.129	0.195	-	-	0.151	0.192	0.169	0.420	0.316	10.250
Night sleeping time	-0.162	0.158	-	-	-0.134	0.156	-0.162	0.352	-0.896	28.316
Napping time	0.001	0.149	-	-	-0.033	0.149	0.112	0.324	-0.220	7.310
CCVD	0.314	0.213	-	-	0.279	0.216	0.670	0.625	0.719	22.771
IADL	0.164	0.173	-	-	0.091	0.172	0.162	0.378	-0.319	10.310
Depression	0.146	0.153	-	-	0.150	0.153	0.009	0.313	0.533	17.037
**TVCs → Cognitive Functions**										
$T1_{(Marriage)}$ → T1	<0.001	0.086	-0.023	0.103	0.014	0.089	-0.029	0.097	-0.020	0.105
$T2_{(Marriage)}$ → T2	0.045	0.062	0.028	0.066	0.056	0.065	-0.023	0.071	0.056	0.073
$T3_{(Marriage)}$ → T3	0.017	0.047	0.065	0.048	-0.017	0.049	0.051	0.054	0.023	0.051
$T4_{(Marriage)}$ → T4	0.076	0.055	0.144	0.059	0.056	0.060	0.061	0.067	-0.009	0.070
$T1_{(Night\ sleeping\ time)}$ → T1	0.022	0.082	0.018	0.095	0.020	0.083	0.028	0.097	-0.040	0.102
$T2_{(Night\ sleeping\ time)}$ → T2	0.046	0.052	-0.024	0.055	0.067	0.054	-0.008	0.060	0.106	0.059
$T3_{(Night\ sleeping\ time)}$ → T3	0.049	0.046	0.078	0.050	0.001	0.049	0.008	0.056	0.028	0.057
$T4_{(Night\ sleeping\ time)}$ → T4	-0.008	0.041	-0.050	0.046	0.016	0.045	-0.022	0.051	0.022	0.055

$T1_{(Napping\ time)} \rightarrow$ T1	-0.040	0.076	-0.010	0.089	-0.048	0.078	0.008	0.090	-0.037	0.096
$T2_{(Napping\ time)} \rightarrow$ T2	0.004	0.048	-0.055	0.051	0.023	0.050	-0.002	0.056	0.040	0.055
$T3_{(Napping\ time)} \rightarrow$ T3	-0.037	0.044	-0.018	0.048	-0.020	0.047	-0.018	0.053	-0.058	0.055
$T4_{(Napping\ time)} \rightarrow$ T4	-0.015	0.041	0.002	0.046	-0.011	0.045	-0.033	0.051	0.036	0.525
$T1_{(CCVD)} \rightarrow$T1	0.079	0.114	-0.256	0.137	0.084	0.117	0.145	0.132	-0.003	0.142
$T2_{(CCVD)} \rightarrow$ T2	-0.009	0.073	**-0.203****	0.078	0.017	0.076	0.054	0.084	-0.019	0.086
$T3_{(CCVD)} \rightarrow$ T3	-0.044	0.058	**-0.206****	0.060	0.005	0.061	-0.002	0.068	-0.007	0.067
$T4_{(CCVD)} \rightarrow$ T4	-0.012	0.059	-0.073	0.063	-0.006	0.064	0.033	0.071	0.030	0.075
$T1_{(IADL)} \rightarrow$ T1	0.078	0.086	0.121	0.102	0.015	0.088	0.106	0.102	-0.093	0.109
$T2_{(IADL)} \rightarrow$ T2	-0.010	0.054	0.111	0.057	-0.010	0.056	-0.052	0.063	-0.118	0.062
$T3_{(IADL)} \rightarrow$ T3	0.034	0.047	0.056	0.050	-0.002	0.050	0.006	0.055	-0.028	0.057
$T4_{(IADL)} \rightarrow$ T4	**0.121****	0.045	-0.076	0.050	-0.081	0.050	**-0.142***	0.055	0.073	0.060
$T1_{(Depression)} \rightarrow$ T1	0.005	0.077	0.017	0.091	<0.001	0.079	0.012	0.092	-0.006	0.098
$T2_{(Depression)} \rightarrow$ T2	-0.017	0.049	-0.040	0.052	-0.010	0.051	-0.051	0.058	0.105	0.057
$T3_{(Depression)} \rightarrow$ T3	**-0.110***	0.043	**-0.144****	0.047	-0.038	0.047	**-0.158****	0.052	0.007	0.055
$T4_{(Depression)} \rightarrow$ T4	**-0.146****	0.043	**-0.138****	0.047	**-0.133****	0.046	**-0.103***	0.052	0.030	0.056
**Fit indexes**										
χ^2	114.388		**116.740****		**117.264****		75.035		94.067	
df	79		79		79		79		79	
CFI	0.933		0.900		0.903		1.000		0.880	
TLI	0.890		0.835		0.840		1.054		0.803	
RMSEA	0.037		0.038		0.039		<0.001		0.024	
SRMR	0.018		0.023		0.019		0.018		0.017	

[a] Standardized score. Bold: statistically significant (* p<0.05, ** p<0.01, *** p<0.001, uncorrected).
CCVD: number of Cardiovascular and Cerebrovascular Diseases; IADL: Instrumental Activities of Daily Living.
A: Univariate LGCM, to investigate the trajectories of cognitive function across 4 waves.
B: Conditional LGCM, to investigate the trajectories of cognitive function and its association with Baseline Covariates (BCs) including age, gender, education, marriage, napping time, night sleeping time, chronic disease, drinking, and smoking in 2011, and Time-Varying Covariates (TVCs) including marriage, napping time, night sleeping time, chronic disease, IADL, and depression across 4 waves.
